# Biopolitics of othering during the COVID-19 pandemic

**DOI:** 10.1057/s41599-022-01435-7

**Published:** 2022-11-14

**Authors:** Dušan Ristić, Dušan Marinković

**Affiliations:** Faculty of Philosophy, University of Novi Sad, Novi Sad, Serbia

**Keywords:** Sociology, Cultural and media studies

## Abstract

The COVID-19 pandemic as a global threat caused the introduction of different biopolitical measures accompanied by discourses on otherness, including xenophobic, racist, nationalist, or new orientalist discourses. The aim of this research is to map discourses on otherness during the COVID-19 pandemic. Our general hypothesis is that, despite the differences in social and cultural contexts, discourses on otherness generated during the pandemic legitimized biopolitical actions and/or measures in addition to exacerbating social, political and cultural differences. The research is based on a semi-systematic approach to literature review using Situational Analysis and Sociology of Knowledge Approach to Discourse. In conclusion, we discuss the impact of discourse studies in the context of the global emergency.

## Introduction

While COVID-19 is new to science as a disease, xenophobic and racist reactions and the processes of othering in pandemics are anything but novel. The “history of ethnic and racial scapegoating for disease is long, with accusations against Jews for fourteenth-century plague, Irish Catholics for early nineteenth-century cholera, and Italians for early twentieth-century polio, to name a few” (Klingberg, 2020, p. 3).

The effects of the “virus-focused” response raises among others, the question of “biopolitics and biosecurity, though they also concern questions of race, ethnicity, and nationalism” (Klingberg, 2020, p. 5). Researchers in social sciences have already raised the concern that “in a global politics characterized by racialized inequality, pandemics such as COVID-19 exacerbate the marginalization” (Dionne and Turkmen, 2020) and othering. Our research aim and contribution are to: (1) situate the current pandemic in a context of the *biopolitics of othering;* (2) map the worlds/arenas where discourses on othering emerge and social actors that launch them; (3) discuss and explain the implications of the *biopolitics of othering*, particularly in the context of the pandemic. These aims are broken into next tasks: the analysis of the discourses on othering found in the research articles published during the COVID-19 pandemic via *Research Gate* platform; making of the *social worlds/arenas* map in accordance with the methodological procedure in Situational analysis; interpretation of results with the Sociology of Knowledge Approach to Discourse framework.

## Otherness, pandemics, and biopolitics

### Otherness

It is about series of complex social practices, sociological questions, and reproduction of social boundaries. In our approach, Otherness is contextualized within pandemic and explained through an analysis of the *biopolitics of othering*.

Iver Neumann recalls that, from Durkheim to Levi-Strauss, it was recognized that “the lineation of an ‘in-group’ must necessarily entail its demarcation from a number of ‘out-groups’” and that “demarcation is an *active* and ongoing part of identity formation” (Neumann, 1999, p. 4). In other words, the creation of social boundaries is an “a priori ingredient” of the processes of group dynamics and identity formation.

According to *The Dictionary of Human Geography*, “the Other is what is excluded from the Self, and which, thanks to that exclusion, constitutes the limits of the Self” (Gregory et al., 2009, p. 515). Duality or the binary opposition Self/Other is one of the key issues in Postcolonial Studies (Sharp, 2009). In one of the most influential studies in the field, *Orientalism*, Edward Said (1979) demonstrated how knowledge about Others is produced through discursive practices and constructions. It was a project of making boundaries, worlds and divisions in these worlds to “Us” and “Them/Others”.

Michel Foucault’s research into the “small worlds” of microphysics of power/knowledge brought about the relevance of marginalized and dangerous Others (Foucault, 1995; 2006). Furthermore, he demonstrated why and how statistically negligible, socially absent, marginalized and politically excluded social actors are not scientifically insignificant. On the contrary, as Tim Cresswell instructively summarizes: “Marginal, grotesque, extraordinary elements and events in society are interesting in themselves, but they are more interesting when we examine the role they play in defining the ‘normal’, the classical, the dominant. The center could not exist without the margin” (Cresswell, 1996, p. 149). The point is: “What is socially peripheral may be symbolically central” (Stallybrass and White, 1986, p. 23).

Anthropology as the science *par excellence* concerned with *differences* was faced with the question of Otherness “since long before that word was spelled with a capital O” (Sax, 1998, p. 292). In that sense, “research on Otherness is not just about the discovery of faraway or distant cultures and territories, but it is about our immediate and close Other” (Marinković et al., 2017).

### Pandemics

They are not just medical, but social phenomena as well. By definition, they affect the society at large. Furthermore, the fact that in pandemics a virus can affect anyone anywhere, “but potentially impacts certain groups more than the others due to different living conditions and material circumstances” (Ward, 2020, p. 4), makes this claim more convincing (Ristić et al., 2020; Hawkins, 2020). For example, due to the current pandemic of COVID-19, “Black Americans and other historically disadvantaged groups experience infection and death rates that are disproportionately high for their share of the total population” (Pelizza, 2020, p. 1). Despite its biological nature pandemic “also impacts strongly the socio-cultural dimension” (Moreno Barreneche, 2020, p. 20). This is why the virus or pandemic itself could not be considered “a major global threat without a discursive environment” (Moreno Barreneche, 2020, p. 20).

When it comes to a social crisis and a public health threat like a pandemic, people often tend to “resort to othering—dissociating themselves from the threat and blaming others—other countries, foreigners, stigmatized groups or other minorities, which helps reduce the powerlessness experienced during the crisis” (Eichelberger, 2007). The threat of disease in particular can lead to the stigmatization of different out-groups and that often inspires violence (Joffe, 1999; Li and Nicholson, 2021). This is a consequence of “discursive nationalism” (Bieber, 2020) and blaming of specific groups, identified as carriers or transmitters of disease. For example, it is the rhetoric on the “Chinese virus” or the “Kung Flu”, which contributed to an atmosphere of anti-Chinese and anti-Asian sentiment and attitudes, even attacks in the US (Reny and Barreto, 2020; Richter et al., 2021). Furthermore, during the current pandemic of COVID-19, "some populists and autocrats have blamed minorities or migrants and other so-called outsiders, creating a link between the disease and specific population groups" (Bieber, 2020, p. 18). The ongoing pandemic has once again put to the test the patterns of social relations, but also unveiled the fragility of human bonds, and introduced “new forms of ignorance and exclusion” (Marinković and Major, 2020).

Throughout history, pandemics “have led to stigmatization, prejudice, ‘othering’ and blame” (Banerjee et al., 2020, p. S102). According to Pelizza, the “yellow fever case constitutes a textbook example of how narratives of disease can eventually enact narratives of alterity” (2020, p. 3). Another example is the Bubonic Plague of the thirteenth century, when the Catholic Church blamed the Jews for “poisoning the water and spreading the illness” (Banerjee et al., 2020, p. S103). The example of Irish immigrants over a century ago in the United States also testifies how impoverished immigrants were stigmatized as the bearers of cholera, whereas tuberculosis was dubbed “the Jewish disease”; in the fifteenth century syphilis was referred as the “French pox” by the English, *morbus Germanicus* by the French”, etc. (Li and Nicholson, 2021, p. 5). One more “classical example” is that of leprosy, which has “traditionally been a disease of stigma, hate and marginalization”, where the affected were usually considered “sinners” (Banerjee et al., 2020, p. S104). The case of othering has been noted also for the influenza pandemic from 1918–1919, referred as “the Spanish flu”. Discrimination and othering against migrants, foreigners and other types of Others in “emerging infectious disease outbreaks continued throughout the twentieth century” (Dionne and Turkmen, 2020, pp. E217–E219).

The groups most affected by othering include healthcare workers, people who have recovered from COVID-19, people from a lower socio-economic status, or those with particular ethnic, racial or religious identities (Schmidt et al., 2020). The COVID-19 pandemic, just as previous ones, demonstrated the same need for blaming and scapegoating, searching for the “responsible” groups as carriers of the virus.

Examples from the past and examples of today already led some researchers to conclude that current pandemics “exacerbate social inequalities, and further marginalize already marginalized groups” (Dionne and Turkmen, 2020, p. E214). The processes of “marginalization, blame, and stigma are more contagious than the virus itself” (Banerjee et al., 2020, p. S102).

### Biopolitics

If we follow the approach of Michel Foucault, who coined the term *biopolitics* (Foucault, 2000), then we recognize that it refers to different practices and technologies established by authorities in order to protect the lives of citizens. Biopolitics denote the practices from the domains of public policies related to health, migration, asylum, and disease prevention, but also legal and political measures related to the development of biotechnology and medical research (Lemke, 2011). Its goal is always to protect the health of the population. However, as Gambetti (2020) writes, “the decision concerning which portions of the population are to be exposed, when, and to what extent” has everywhere been a political decision, not a medical one (Hatzikidi, 2020).

Biopolitical measures introduced in the COVID-19 pandemic range from centuries old, such as the so-called “lockdown” or quarantine to the face masking, physical distancing, suspension of travel, various restrictions on mobility and border closures. The effects of the “virus-focused” responses also raise questions of biopolitics and biosecurity (Klingberg, 2020, p. 5). Biopolitical measures include the spaces like “the island detention center, the cruise ship, the airport, the train station, the quarantine hotel, and the home” (Lozanovska et al., 2020, p. 417). Moreover, all these measures required people to adjust their daily lives (Méndez Fierros and Reyes Piñuelas, 2021, p. 5).

With the increase in numbers of people affected on a global scale, and with the absence of a vaccine (at least for the majority of global population), widespread myths, misconceptions, conspiracy theories and other kind of narratives, stories and fake news appeared in the media, especially social media. That is why the World Health Organization recognized that the COVID-19 pandemic and the global public health responses have been “accompanied by an ‘infodemic’, which is an over-abundance of information—some accurate and some not” (WHO, 2020, p. 13).

Biopolitical measures often “correspond[s] with disadvantaging factors in people’s lives, which can produce multilayered forms of discrimination” (Röthmuller, 2021, p. 3). The fear of the disease also increased social exclusion, stigmatization and othering, which a number of studies in Asia, Northern and Southern America, Africa and European countries already revealed (Schmidt et al., 2020).

Similar to referred research studies in this paper, we assume that pandemic fuel othering processes because of people’s fear, need for security, safeness and sense. Othering is manifested in the discourses and practices of blaming and the (re)creation of imaginary boundaries, but also through the imposing of physical borders and social distancing.

A discourse of othering mediates cultural and social positioning (including knowledge, attitudes and practices), concerning those deemed responsible for transmitting the virus. Furthermore, the discourses and practices of othering could be identified and recognized through the two patterns: “First relates to developing narratives whose aim is scapegoating of migrants, refugees, asylum seekers, and minority population” (Chung, 2020; Ivić and Petrović, 2020). This way, othering establishes a sharp binary opposition between “Us” and all others that can be a threat to health and security. The second pattern, also according to Chung (2020), can be identified in the mobility restrictions worldwide. Although biopolitics mostly has a legal base and could probably be legally justified, the problem arises when it comes to the limitation of citizens’ rights, isolation, discrimination, marginalization, xenophobia, violence, etc.

In summing up theoretical framework, our key concepts are as follows. We accept the definition of othering as “a discourse that employs a power ‘to construct particular subject positions for ‘us’ by designating a certain category of people as ‘them’ (the Other)” (Liu and Self, 2020, p. 463; Ivić and Petrović, 2020, p. 423). Biopolitics encompass practices and technologies of power/knowledge and all measures introduced by authorities (during the pandemic) in order to protect the health of the population. Others, especially in the pandemic, are all those “types of people” and social groups that are symbolically or physically excluded from the community and labeled as potential or actual threat to health (from healthcare workers to the ethnic/national/racial communities).

Our hypothesis is that discourses and practices of othering during the COVID-19 pandemic should be understood beyond the politics, in terms of biopolitics. We are going to test this hypothesis through the analysis of literature and research studies conducted during the COVID-19 pandemic worldwide.

## Research methodology

The objective of this research is to summarize the representations of othering in the analysis of the social scientific articles published during the COVID-19 pandemic. Furthermore, the focus on the discursive aspects of othering is for the sake of recognition and demonstration of the relevance of this kind of research, especially in the context of pandemic and similar emergencies.

Our epistemological framework is interpretative, and methodological framework is qualitative. In data/articles collection, analysis and processing, we used a few research techniques and methodological frameworks. In coding and processing the data, Content analysis and Situational analysis were used. This kind of research methodology allows us to “draw together studies of discourse and agency, action and structure, image, text and context, history and the present moment—to analyze complex situations of inquiry broadly conceived” (Clarke, 2005, p. xxii). An additional reason for choosing this theoretical-methodological package was because it allowed us to connect it with a particular type of discourse analysis approach called Sociology of Knowledge Approach to Discourse (SKAD) used for the interpretation of data (Keller, 2012).

The sample for this research is composed of articles found at the *Research Gate* platform and their COVID-19 research community page.^1^ The articles are listed at the end of this article, in the “*Analyzed corpus*” *section*. The platform was chosen because during the pandemic it was one of the academic networks that collected relevant and most recent research on COVID-19. We used three keywords in search for the adequate articles for the analysis. Those were: COVID-19, Other, Otherness. All three were used because they covered a bit more articles than in case of using Other and/or Otherness. There were twenty-seven articles that fit the keywords in search. Twenty articles were selected out and analyzed, to avoid redundancy and similarity in analyzed topic. An additional criterion in choosing the articles for the sample was that the text was published in English and throughout 2020/2021, during the COVID-19 pandemic.

A *literature review* was used as a research methodology. It can broadly be described as “a more or less systematic way of collecting and synthesizing previous research” (Snyder, 2019). This kind of research is used as a “firm foundation for advancing knowledge and facilitating theory development”, but also as a way to “synthesize research findings”, “show evidence” or “uncover areas in which more research is needed” (Snyder, 2019, p. 333). Literature review as a research methodology except in case when it is complete and systematic, does not offer the possibility to encompass all cases. That is why we used semi-systematic approach. It is adequate “for topics that have been conceptualized differently and studied by various groups of researchers within diverse disciplines and that hinder a full systematic review process” (Snyder, 2019, p. 335). Finally, it offers possibility to map the field, and eventually create an agenda for further research.

Situational analysis is adequate for this kind of research since it helps researchers to map the field of research or the so-called “situation”. Clarke (2005) suggested three kinds of maps characteristic for this “cartographic approach”. One of the maps we used in this research. It is the so-called “*Social worlds/arenas map*”, which “lay[s] out the collective actors, key nonhuman elements, and the arena(s) of commitment and discourse within which they are engaged in ongoing negotiations—meso-level interpretations of the situation” (Clarke, 2005, p. xxii). As pointed by Clarke (2005, p. 10), “the particular power of the social worlds/arenas framework is that because social worlds are “universes of discourse’, the framework goes beyond ‘the usual suspects”—the usual highly bounded sociological framings of organizations, institutions, and even social movements. These are displaced by a more open, fluidly bounded, discourse-based framing of collective action”.

In the analysis and interpretation of articles we used the Sociology of Knowledge Approach to Discourse (SKAD) (Keller, 2012). Within this research program, discourses are considered as “historically situated real social practices, not representing external objects, but constituting them” (Keller, 2012, p. 53). Thus, with SKAD it is possible to recognize how construction, objectivization, communication and legimitization of “meaning structures” appear in the institutional settings or social arenas, including the analysis of their social effects (Keller, 2012, p. 59). Similar to Situational analysis, discursive fields in SKAD are recognized through the *social arenas*, which constitute themselves around contested issues, controversies, problematizations, etc. (Keller, 2012, p. 61). However, this approach unlike Situational analysis and Grounded Theory, “does not aim to explore particular ‘situations and (inter)actions’, but ‘discourses’” (Keller, 2011). That is why SKAD and Situational analysis are “different although somehow complementary strategies in sociology” (Keller, 2011, p. 46; 2013). Furthermore, we recognized additional quality of SKAD, comparing to other interpretative approaches. Namely, *discourse* in SKAD is not just something “subjective” or produced by subjects, but is also something to be found in documents or articles. We approach discourses as “effects” and try to map the “discourse-oriented context”.

Finally, while the *literature review* as a research methodology helped us to determine the sample, SKAD and Situational analysis helped us to: (a) find the “common denominator” and create a “situation” out of the heterogeneous corpus of research (articles); it is because two complementary approaches can be applied to material or corpus consisting of different sources and methodologies (Keller, 2013); (b) apply the type of discourse research that is not “a pure” textual or linguistic research, but also interested in social context and the production of meaning and knowledge (Keller, 2013); (c) recognize different social actors, institutional settings, and “actants”; (d) map them all as the elements of the particular situation (“discursive field”).

The limited range of use of these methodological tools in our case is a matter of the sample and corpus. Additional argument for choosing Situational analysis is our focus on “textual material”. This approach establishes no difference between “text” and “context”. It is *the situation* that reveals both text and context. Moreover, while it possibly limits the scope of analysis, it still stands within the aims and tasks of this research.

Based on this framework, the sample was collected and the qualitative data analysis software Nvivo 12+ for Windows was used for the categorization, coding and analysis of data. During the coding process, an initial code tree has been developed, with additional sub-codes. The initial categorization of data and coding is guided by the keywords: COVID-19, Other, and Otherness. Furthermore, coding process followed the general aim of the research: to map the discourses on Others related to Covid-19 pandemic. The code list includes the following: Biopolitics and politics of life, Borders, Covid-19 virus, Discrimination, Emotional policy discourse, Fear, Nationality, Othering, Others (including sub-codes: “African communities”, “Black people”, “Chinese virus”, Ethnic minorities, “Good” vs. “Bad” citizens, “Latinx people”, Migrant workers, Outsiders, Poor people, Strangers, “Us” vs. “Them”, Virus as Other, “White European”, Workers, Vulnerable populations), Public (Health) policy, Racial discourses, Risk discourses, Social actors—Others, Social and Health inequalities, Social aspects of pandemic, Xenophobia.

## Results and discussion

**Fig. 1:**
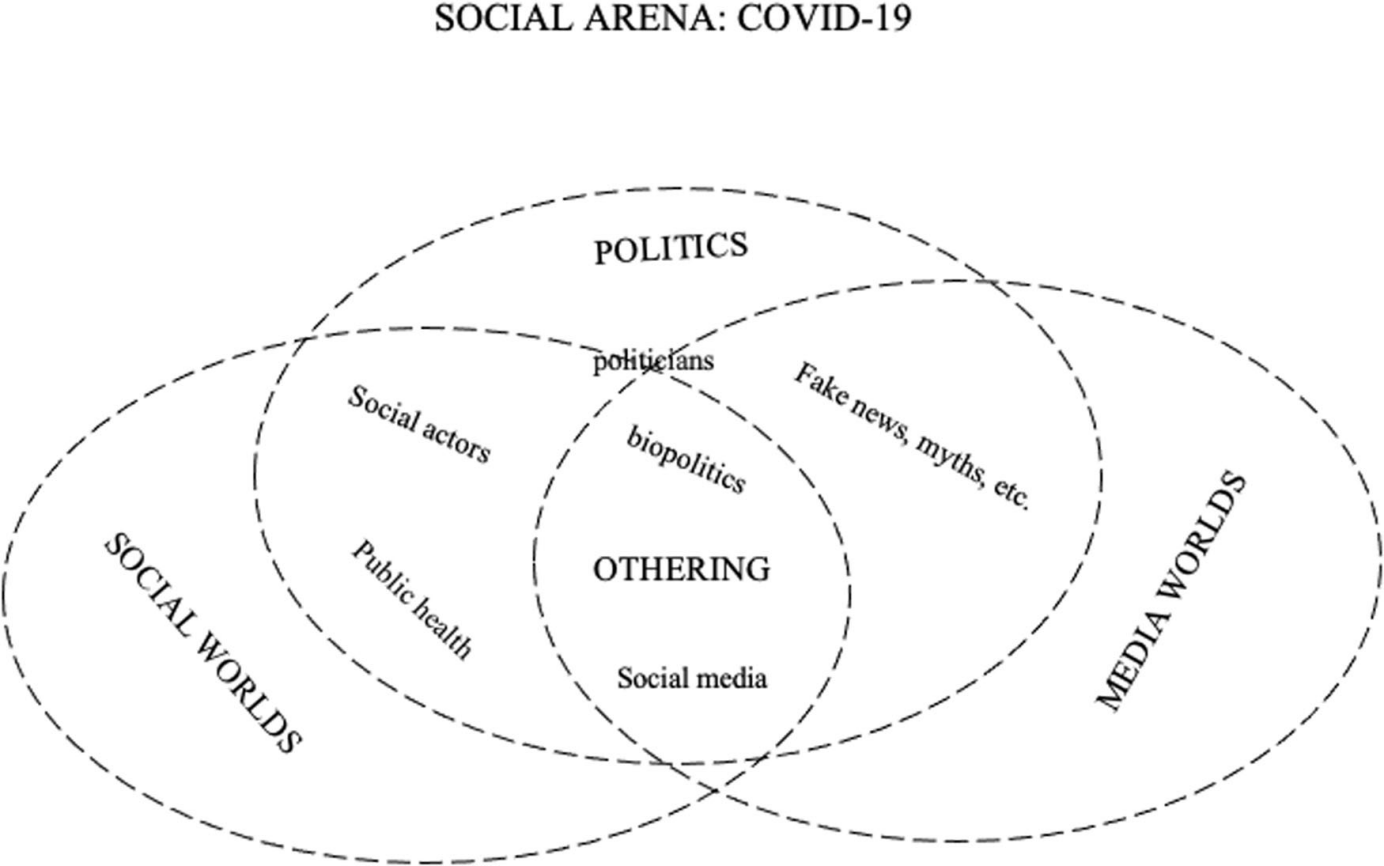
Social worlds/arenas map: *COVID-19, Biopolitics and othering*.

When it comes to *Social worlds/arenas*, we identified three crucial for understanding the emergence of discourses and practices of othering. These are *social*, *political* and *mediatized* worlds/arenas. A visual representation is given in Fig. 1.

S*ocial worlds* in this research is to be understood through the social actors and their discursive strategies in the first place. The analysis of the research articles shows that social actors and groups are included in the reproduction of othering as a reaction following the situation of danger and fear. Health protection in the COVID-19 pandemic evokes “symbolic dimensions related to the social representations of ‘others’ that are emotionally driven by fear or mirror the vulnerable self, activating the othering–otherness process” (De Rosa and Mannarini, 2021).

In this process of the creation of the social representation of Others, two basic strategies were identified: *anchoring* and *objectification*. They include making sense of “unfamiliar events through a connection to past familiar experiences and objects” and “giving abstract concepts (such as a virus) concreteness” (De Rosa and Mannarini, 2021). When it comes to the discursive construction of collective actors, researchers identify processes and mechanisms of actorialization, generalization and axiologization as well (Moreno Barreneche, 2020, p. 2). For example, actorialization means that “in the case of the COVID-19 outbreak, a number of actors have been constructed in discourse. On the side of the heroes, ‘the workers of the health system’, an imagined actor whose members are unknown from an individual perspective and whose bravery is in several countries recognized on a daily basis with a collective round of applause” (Moreno Barreneche, 2020, p. 26). The following process of generalization means the following: “Collective actors, then, in their quality of being discursive constructions based on the attribution of a homogeneous identity to an imagined group, gain life as opposed to other actors, also imagined, based on the dichotomy anchored on the pronouns ‘We’ and ‘They’” (Moreno Barreneche, 2020, p. 26). In addition: “When dealing with collective identities, however, the most interesting semiotic mechanism involved in the social construction of the Other is axiologization, which consists in the attribution of specific value and normative connotations to the collective actors that have been created in discourse” (Moreno Barreneche, 2020, p. 26). These mechanisms are usually followed by xenophobic and racist attitudes and their public manifestation. The cases and examples are evident in countries all around the world (Moreno Barreneche, 2020).

We recognize that sometimes it was difficult for researchers to determine what kind of social groups or communities were particularly prone to the othering discourses and practices. Usually, research studies tackle the problem “in general” and eventually identify the examples in the media reports. That is why we conclude that the most important social arenas are media and politics (politicians). In these mediatized and “politicized” worlds/arenas, many types of Others emerged. They include: Migrant Workers/Strangers (Canada), “Bad” Citizens (Brazil), Race-Ethnicity-Nationality (Hungary, Italy, China, U.S.), Outsiders—domestic and foreign (China), Asian Americans (U.S.), African communities and Black people (China), Latinx people (U.S.), Virus-as-Other, Religious Other, healthcare workers, people who are recovering from COVID-19, people who have COVID-19, lower socio-economic groups, rich people, and White people.

To sum up, within the context of the COVID-19 pandemic, different Others have been constructed in discourses, “in order to articulate a—narrative—explanation for the spread of the virus”, because of the need to find a “sort of villain”, i.e., an actor to be blamed for the circumstances (Moreno Barreneche, 2020, p. 2). On the other side, there are cases and research studies in which the results show a high level of awareness and knowledge of the transmission and prevention of disease. Such is the example from South Africa (Schmidt et al., 2020). Researchers investigating COVID-19 knowledge, attitudes, and practices (KAPs) of residents in Hubei, China, found similar results as well (Schmidt et al., 2020, p. 2).

Expectedly, the key “nonhuman element” in the production of Others are *mediatized worlds/arenas*. The production of Others in media is often characterized by simplistic and “stereotypical dichotomic attributions such as own/alien, good/bad, or morally superior/inferior that ultimately create an ‘us vs. them logic’” (Richter et al., 2021). Media create the specific kind of “bond of belonging” in such a manner that “may refer to a variety of ingroups, such as a certain political group or a religious current or a class, race, or ethnicity” (Richter et al., 2021).

Discourses of othering produced by media contribute to the rise of xenophobia, racism and other forms of anti-social behavior. According to the UN Secretary-General Antonio Guterres, this pandemic is already recognized as a “human rights crisis” that has led to a rise of ethno-nationalism, populism and authoritarianism (Ivić and Petrović, 2020, p. 427). A similar report and recognition come from the Human Rights Watch (Donmez, 2020; Ivić and Petrović, 2020). Furthermore, hundreds of hate speech reports have been identified in the UK, South Korea, Japan, France, Spain, Russia, Australia, Ethiopia, Kenya, etc. One study from San Francisco State University tracked racism and xenophobia in media reports about the current pandemic (Ivić and Petrović, 2020, p. 427). A French regional paper *Courrier picard* warned of a new “Yellow Alert“; German *Der Spiegel* wrote about the crisis “Made in China“; The *Wall Street Journal* headlined about the “China Is the Real Sick Man of Asia”; and the “Chinese virus“ became the term of choice for many politicians (Klingberg, 2020, p. 3).

Depending on the social and cultural context, blame and othering were directed at different kinds of Others. In Argentina and Uruguay, for example, where the virus was imported from travelers arriving from Europe, the spread of COVID-19 was associated with a specific social class. The viral content on the social media fueled the hypothesis that COVID-19 had been introduced in Uruguay by “the posh” (Moreno Barreneche, 2020).

Social media are a telling example of how *infodemic* is entwined with the pandemic. Researchers tracked social media with the attempt to identify myths connected with COVID-19 and they range from conspiracy theories about the origin of the virus, to misinformation that “warm weather kills the virus” (Schmidt et al., 2020, p. 2). A diffusion of pseudoscientific accounts, such as ideas of a specific “minorities’ immunity” or other kinds of “unsubstantiated narratives” (Pelizza, 2020) fueled the fake news and scarcity of data. Owing to the lack of medical knowledge and data or timely information, insinuations and misinformation flourished, e.g., that “people of color may be immune to the coronavirus because of melanin” (Williams, 2020), or of “blood genetics composition of sub-Saharan Africans” (Teresa, 2020), or the widely diffused thread that “immigrants do not get sick of COVID-19 thanks to their anti-tuberculosis vaccine” (Meli, 2020; but see: Pelizza, 2020, p. 2).

What researchers already recognized is that myths, misconceptions and fake news can “hamper efforts to mitigate the transmission of the disease” (Schmidt et al., 2020, p. 14). That is why the identification of conspiracy theories, myths, fake news and other misconceptions is crucial during the pandemic. It can help experts in public health policy to prevent the spreading of virus and open the space for accurate and verified information (Larson, 2020).

The social world/arena whose “main actors” are identified in the research studies during the pandemic as those who generate the narratives and discourses on Others are *politicians*. Generally speaking, we recognized two “discursive strategies” of politicians in the analyzed articles. The first is the *production of narratives* about Others by blaming, scapegoating, demonizing and marginalizing (strangers/foreigners, tourists, members of other ethnic/national group or any kind of minority, etc.). The second strategy is *naming* the pandemic.

For example, Matteo Salvini, a senator and former deputy prime minister in Italy, said that his government was “irresponsible to allow a rescue vessel with 276 African migrants to dock in a Sicilian port, although at the time of his remarks there was only a single case of the virus reported on the African continent” (Dionne and Turkmen, 2020, p. E221). In North America, former U.S. president Trump spoke about the “Chinese virus” (Richter et al., 2021). A nationalist agenda has been strengthened in many countries, including the Middle East and North Africa, where “homogenizing and demonizing *othering* was detected in particular in the cases of Yemen and Egypt, but also Iraq” (Richter et al., 2021, p. 3). Politicians were also the actors who spread fake news or unverified information during the pandemic. In India for example, Uttar Pradesh chief minister Yogi Adityanath claimed that “migrant workers are strong man used to ‘sweat’ and as such recover much faster from COVID-19 than ‘normal persons’” (Pelizza, 2020, p. 2).

A particularly important element in the othering processes is the “politics of naming”. It can “shape how media covers the crisis and how citizens understand disease spread and vulnerability to infection” (Dionne and Turkmen, 2020, p. E225). We know about the example of “Spanish flu” from the beginning of the previous century, where naming was also used as a “rhetoric strategy” that promotes association between disease and a foreign country. In the current pandemic, as the number of infected people rose in the US and around the world in early 2020, “conservative elites in the US racialized the pandemic, referring to the coronavirus as the ‘Chinese flu’ or the ‘Wuhan virus’” (Reny and Barreto, 2020). Place-naming or associating a pandemic with a social group may be “particularly consequential, even if later media responses shift toward emphasizing safety” (Dionne and Turkmen, 2020). For this reason, back in 2015 the World Health Organization called for a disease epidemic “to be named according to the pathogen rather than a geographical location where initial cases were reported” (Dionne and Turkmen, 2020, p. E225).

In our sample, we found the examples of the *biopolitics of othering* that confirm our initial assumptions. Generally speaking, worldwide biopolitical measures were accompanied by a rhetoric characterized by the fear of the foreign ‘other’. The introduction of these measures reveals how they can, on the one side, protect the economy, society and health of people in the country. However on the other, they make certain groups or populations vulnerable. There is the example in Canada, where fear and anxiety regarding the spread of the virus brought “into focus longstanding, almost always racialized, representations of ‘dangerous foreigner’, which in turn enables restrictive policies that vulnerabilize migrant workers” (Larios and Paterson, 2021). Another example comes from Brazil, where president Bolsonaro’s “response to the pandemic marked a public transition from valuing certain kinds of lives as opposed to others, to a general contempt for human lives” (Hatzikidi, 2020, p. 72). In his “public safety agenda” othering is promoted as a duality between *cidadãos de bem* (righteous citizens) and *bandidos* (criminals). This “Manichean discourse reduces complex realities and structural inequality to a battlefield”, but is also followed by the attempt of installation of the biopolitical measures. During the May of 2020, the president of Brazil accompanied by the Minister of the Economy and businessmen “marched” to the Supreme Court to urge them to “retract an earlier decision, which allowed state and local authorities to implement social distancing measures as they saw fit” (Hatzikidi, 2020, p. 72). There is also the example of generating disinformation, in the case of Mexican federal government headed by President Lopez, where “heavy criticism of the safeguards was adopted by people and their compliance with the mandated safety measures, as well as a rejection of Americans crossing into Mexico” (Méndez Fierros and Reyes Piñuelas, 2021, p. 12). Subsequently, Otherness was articulated in relation to the virus and this was followed “by words calling for action: close, [not to] cross, closed” (Méndez Fierros and Reyes Piñuelas, 2021, p. 14).

We also found some different cases, where media did not enforce a discourse on the nation being threatened by “external forces”, such as in Iraq. Their media “emphasized a more global perspective and asked about the immoral Other in world politics” (Richter et al., 2021). Furthermore, the Omani case show how media “relied strongly on the importance of national citizenship as a marker of an in-group that needs to be protected, reinforcing a global trend as Bieber (2020) had predicted” (Richter et al., 2021).

To sum up, in line with the goals and objectives of this research, analysis of the articles identified three important social effects of othering. First, our sample shows that discourses of othering during the pandemic contribute to the rise of different forms of anti-social behavior (xenophobia, racism, violence, etc.). Second, we find that different kind of Others emerge, depending on the social and cultural context. Third effect is *biopolitical*, and refers to the general hypothesis of our research. While we find the studies that recognize the importance of the (discursive) othering during the pandemic of COVID-19 (Richter et al.,^ 2021^), we do not find any research that opens the question about the discursive legitimization of biopolitical measures.

Although further study is warranted, we find the examples (Canada, Brazil, Mexico) where discourses of othering legitimize biopolitical actions and/or measures. Discourses on Others (in media particularly), legitimize decision-makers (namely politicians) to implement biopolitical measures, especially not “popular” ones. Moreover, these measures often produce multilayered forms of discrimination, as Röthmuller (2021) indicates. That is an important argument in favor of the general hypothesis of this research.

## Conclusion

The main finding of our research is that the processes of othering are ubiquitous during the pandemic, one way or another. Although we identified cases and countries in which the othering processes are connected with the biopolitical measures, this connection is not clearly demonstrated, through the cause-consequence relation. Lessons learnt from previous pandemics might prove to be helpful to understand how different kinds of othering facilitate the transmission of virus. During the 2014 Ebola outbreak for example, “CDC director Tom Frieden argued against the effectiveness of lockdown for both epidemiological and social reasons, that casting too wide a net, such as invoking travel bans, would only provide illusion of security and would lead to prejudice and stigma” (Klingberg, 2020, p. 9). Furthermore, “HIV-related stigma has been cited as one of the most enduring barriers to ending HIV pandemic” (Schmidt et al., 2020, p. 3).

The necessity to address and research further the question of othering in pandemic stems from the fact that it may help guide the virus-focused epidemiological responses.

## Data Availability

All data analyzed are contained in the article.

## Footnotes

1 https://www.researchgate.net/community/COVID-19
